# Fast SAR Image Change Detection Using Bayesian Approach Based Difference Image and Modified Statistical Region Merging

**DOI:** 10.1155/2014/862875

**Published:** 2014-08-28

**Authors:** Han Zhang, Weiping Ni, Weidong Yan, Hui Bian, Junzheng Wu

**Affiliations:** ^1^Northwest Institute of Nuclear Technology, Xi'an 710024, China; ^2^School of Electronic Engineering, Xidian University, Xi'an 710071, China

## Abstract

A novel fast SAR image change detection method is presented in this paper. Based on a Bayesian approach, the prior information that speckles follow the Nakagami distribution is incorporated into the difference image (DI) generation process. The new DI performs much better than the familiar log ratio (LR) DI as well as the cumulant based Kullback-Leibler divergence (CKLD) DI. The statistical region merging (SRM) approach is first introduced to change detection context. A new clustering procedure with the region variance as the statistical inference variable is exhibited to tailor SAR image change detection purposes, with only two classes in the final map, the unchanged and changed classes. The most prominent advantages of the proposed modified SRM (MSRM) method are the ability to cope with noise corruption and the quick implementation. Experimental results show that the proposed method is superior in both the change detection accuracy and the operation efficiency.

## 1. Introduction

Due to the advantage of gathering imagery in all daytime and all weather conditions, synthetic aperture radar (SAR) images are quite valuable for identification of changes that have occurred after a natural or anthropic disaster [1–7]. However, the task of SAR image change detection (CD) suffers from the presence of intrinsic speckle noise. The traditional solution is despeckling with various filters such as the frost filter [4], gamma-MAP filter [5], or the famous nonlocal mean (NLM) filter [6]. Unfortunately, the loss of some details inevitably occurs and also the despeckling procedure increases the time complexity significantly. Consequently, most of the state-of-the-art methods have to make a compromise between the CD accuracy and the operation efficiency. Hence further study of automatic SAR image change detection methods is desired.

Change detection can be divided into two procedures. First is the generation of difference image (DI) with a comparison operator. Second is the classification of DI into two classes: the changed class and the unchanged one. Comparison in SAR images is typically carried out by a log ratio operator [3, 5–10]. The log ratio (LR) DI reduces the multiplicative distortion effects of noise that are common to the two considered images due to speckle and make the statistical distribution of the resulting image depend only on the relative changes between the two acquisitions [7]. Instead of the pixel-based comparison, a statistical similarity measure, cumulant based Kullback Leibler distance (CKLD), is developed based on the evolution of the local statistics of two images [11]. However, the CKLD cannot detect changes where the local statistics stay the same. Besides, it shows poor detail preservation capability. The classification procedure is conducted either by threshold algorithms or by region merging algorithms. The thresholding techniques compute a threshold on the basis of global or local statistics and apply it to either the entire image or only the local area. The mixed Gaussian distribution [8], generalized Gaussian distribution [9], and the bivariate gamma distribution [10] are applied to model the statistical properties of LR difference images. In fact, the amplitude of variation in the observed scenes between two time instances is not predicable. The statistical models mentioned above cannot always describe DI properly.

Different from threshold methods, region clustering methods can incorporate regional features. The problem involves establishing the initial regions and finding reasonable region descriptors. Many existing low level merging methods, such as mean-shift [12], watershed [13], and level set [14], can be used for the initial regions. Several region descripting approaches for high level merging have been proposed for SAR images. A statistically homogeneous region aggregation method [15] applies the coefficient of variation to evaluate the segment homogeneity. Ji and Li [16] introduce superpixels based on independent component analysis as the region feature. A spectral clustering method for region merging is presented in [17] which obtains the globally optimal solutions in a relaxed continuous domain. Hierarchical region growing models [18, 19] have demonstrated efficacy for SAR image region merging. Gong et al. [3] incorporate the MRF models into fuzzy C-means clustering method to exploit statistical correlation of intensity levels among neighboring pixels to decide whether the central pixel is speckle. MRF serves as an opportune tool to introduce local information but is easy to get into local optimum. Besides, the clustering approaches mentioned above all involve iterative calculation which is quite time consuming.

In this paper, we develop a novel fast SAR image change detection method. Taking into consideration of the prior information that the multiplicative speckle noise follows the Nakagami distribution [5, 20, 21], a new comparison operator is deduced within a Bayesian framework. The resulting DI shows bigger statistical separability between the unchanged and changed regions. Speckles are depressed obviously compared with the popular LR. For the classification procedure, the statistical region merging (SRM) method [12] is firstly introduced to SAR image change detection context. The most prominent advantage of SRM is the quick implementation as well as the ability to cope with noise corruption. The region merging procedure follows a particular order in the choice of regions to avoid recursive calculation. The initial region establishment is also escaped. In our modified SRM algorithm (termed as MSRM), a new statistical variable is introduced for merging predication. The merging procedure ends up with two classes, the unchanged class and the changed one, labeled as {*uc*, *cc*}. The main novelty of this paper lies in two aspects. One is the disengagement to despeckling process either with the filtering approach or by incorporating the MRF model, making it quite robust with changes of different characteristics. Second is the fast implementation which is essential for real-time application.

## 2. DI Generation

In SAR images, the reflectivity *R*_*s*_ in site *s* is considered to be corrupted by the multiplicative Goodman's speckle noise model [21]. The pixel amplitudes *I*_*s*_ are modeled independently and identically distributed according to the following Nakagami distribution:

(1) $$\begin{array}{c} p\left(I_{s}\;|\;R_{s}\right)=\frac{2L^{L}}{\Gamma \left(L\right){\left(R_{s}\right)}^{L}}I_{s}^{2L-1}\exp\left(-\frac{LI_{s}^{2}}{R_{s}}\right), \end{array}$$

where *L* is the equivalent number of looks (ENL).

In the context of the Bayesian decision theory [22], the site *s* is assumed to be changed if the following relation holds:

(2) $$\begin{array}{c} p\left(I_{s}^{1},I_{s}^{2}\;|\;R_{s}^{1}=R_{s}^{2}\right)<p\left(I_{s}^{1},I_{s}^{2}\;|\;R_{s}^{1}\neq R_{s}^{2}\right), \end{array}$$

where *I* ^1^ and *I* ^2^ are the first- and second-data SAR images, respectively, and *R* ^1^ and *R* ^2^ are the first- and second-data ground truth reflectivity, respectively.

Inequality (2) can be rewritten as

(3) p(Rs1=Rs2 ∣ Is1,Is2)p(Is1,Is2)p(Rs1=Rs2) <p(Rs1≠Rs2 ∣ Is1,Is2)p(Is1,Is2)p(Rs1≠Rs2),

where

(4) $$\begin{array}{c} p\left(R_{s}^{1}\neq R_{s}^{2}\right)=1-p\left(R_{s}^{1}=R_{s}^{2}\right)\\ p\left(R_{s}^{1}\neq R_{s}^{2}\;|\;I_{s}^{1},I_{s}^{2}\right)=1-p\left(R_{s}^{1}=R_{s}^{2}\;|\;I_{s}^{1},I_{s}^{2}\right). \end{array}$$

Then we have

(5) p(Rs1=Rs2 ∣ Is1,Is2)<p(Rs1=Rs2) ⟹p(Rs1=Rs2,Is1,Is2)p(Is1,Is2)<p(Rs1=Rs2) ⟹p(Rs1=Rs2,Is1,Is2)p(Rs1=Rs2)<p(Is1,Is2) ⟹p(Is1,Is2 ∣ Rs1=Rs2)<p(Is1,Is2) ⟹∫p(Is1,Is2 ∣ Rs1=R,Rs2=R)dR<p(Is1,Is2).

Under independence assumption on *I*_*s*_^1^ and *I*_*s*_^2^, we have

(6) ∫p(Is1,Is2 ∣ Rs1=R,Rs2=R)dR =∫p(Is1 ∣ Rs1=R)p(Is2 ∣ Rs2=R)dR.

Inequality (5) is eventually manipulated as

(7) $$\begin{array}{c} \int p\left(I_{s}^{1}\;|\;R_{s}^{1}=R\right)p\left(I_{s}^{2}\;|\;R_{s}^{2}=R\right)dR<p\left(I_{s}^{1},I_{s}^{2}\right)\leq 1. \end{array}$$

Noting the following equality

(8) $$\begin{array}{c} \overset{\infty }{\underset{0}{\int }}\frac{A}{x^{n}}\exp\left(-\frac{B}{x}\right)dx=AB^{1-n}\Gamma \left(n-1\right), \end{array}$$

we have

(9) ∫p(Is1 ∣ Rs1=R)p(Is2 ∣ Rs2=R)dR =∫0∞4L2L(Is1)2L−1(Is2)2L−1Γ2(L)R2L×exp⁡(−L((Is1)2+(Is2)2)R)dR =4LΓ(2L−1)Γ2(L)(Is1Is2+Is2Is1)1−2L.

Inequality (5) is further brought out as

(10) $$\begin{array}{c} \log\left(\frac{I_{s}^{1}}{I_{s}^{2}}+\frac{I_{s}^{2}}{I_{s}^{1}}\right)\geq \frac{1}{1-2L}\log\frac{\Gamma^{2}\left(L\right)}{4L\cdot \Gamma \left(2L-1\right)}. \end{array}$$

Inequality (10) indicates that decisions can be made based on the change variation index expressed by the left hand of (10) and the threshold expressed by the right hand which only depends on the ENL. Note that an independence assumption is made in the derivation process; we cannot rely on this threshold. However we get a new DI (termed as NLR) incorporating the prior that speckles follow the Nakagami distribution:

(11) $$\begin{array}{c} \text{NLR}=\log\left(\frac{I^{1}}{I^{2}}+\frac{I^{2}}{I^{1}}\right). \end{array}$$

We can see that NLR is quite similar with LR. Later we will find out that NLR is better than LR in the aspect that it enhances the statistical separability between the changed and unchanged regions, which helps improve the clustering accuracy in the following procedure. Besides, the NLR operator is computationally simple.

It is worth noting that a similar DI defined as *η*_*s*_^1^/*η*_*s*_^2^ + *η*_*s*_^2^/*η*_*s*_^1^ (termed as GR) is proposed in [4], where *η*_*s*_^*i*^ represents the estimation of intensity mean in a local homogenous area in site *s*. This difference measure is deduced by a likelihood ratio test under the assumption that the intensity value of SAR image in a homogenous region follows the gamma distribution. We compare GR with NLR in Section 4.

## 3. Modified Statistical Region Merging

According to human perception theory, each image *I* has its corresponding best statistical segmentation I*. The SRM approach turns the problem of image segmentation into the problem of statistical inference. It exhibits a particular blend of algorithms and statistics whose segmentation error is limited from both the qualitative and quantitative standpoints. The order merging strategy gains linear time complexity. By using the average region gray level as statistical inference variable, the merging predicate inequality is deduced.

However, we cannot apply SRM directly to SAR image change detection purposes. First, the region number of SRM result depends on the complexity of the original image, while the CD final map usually contains only two classes, the changed one and unchanged one. So we need to control the merging ending process. Second, with the interruption of speckles, the average gray level cannot identify a region properly by itself. Therefore, some modifications are made.

The classical SRM consists of two merging procedures. The first merging traverses a specific order once, resulting in the primary segmentation map. To handle the occlusions, the merging process is run again on the primary map. Empirically speaking, the DI is segmented into 5 to 10 regions until now. Inspired by [15, 23], where the different land cover types of SAR images are effectively classified by merely using the local mean and variance, a third merging process is defined with the region variance as a new statistical inference variable.

### 3.1. Statistical Inference Inequality

Let I* be the best statistical segmentation map of *I*, the gray level *g* ∈ [0, *G*], and *g* belongs to one of the *Q* different random variables (r.v.) for the whole image. The variation range is [0, *G*/*Q*] for each (r.v.). *Q* indicates the statistical complexity of image *I*. Consider fixed couple regions (*R*, *R*′) of *I*; the observed gray level average of region *R* is $\bar{R}$. The SRM statistical inference inequality for the first and second merging procedure is [24]

(12) $$\begin{array}{c} \left|\bar{R^{\prime }}-\bar{R}\right|\leq \sqrt{b^{2}\left(R\right)+b^{2}\left(R^{\prime }\right)}, \end{array}$$

where $b\left(R\right)=G\sqrt{\left(1/\left(2Q\left|R\right|\right)\right)\ln\left(\left|R_{\left|R\right|}\right|/\delta \right)}$, in which |*R*| is the number of pixels in region *R*, *R*_|*R*|_ denotes the set of regions with |*R*| pixels with |*R*_|*R*|_| = (|*R*|+1)^min⁡(|*R*|,*G*)^, and *δ* is a tiny probability value usually set as *δ* = 1/(6|*I*|^2^). The current region couple (*R*, *R*′) is merged if (12) holds.

Inequality (12) is deduced from the independent bounded difference inequality theorem as follows.

**Theorem 1 .**

Let *X* = (*X*_1_, *X*_2_,…, *X*_*n*_) be a family of *n* independent r.v. with *X*_*k*_ taken values in a set *A*_*k*_ for each *k*. Suppose that the real-valued function *f* defined on Π_*k*_ *A*_*k*_ satisfies |*f*(*x*) − *f*(*x*′)| ≤ *c*_*k*_ whenever vectors *x* and *x*′ differ only in the *kth* coordinate. Let *μ* be the expected value of the r.v. *f*(*X*). Then for any *τ* ≥ 0,

(13) $$\begin{array}{c} \text{ Pr }\left(f\left(X\right)-\mu \geq \tau \right)\leq \exp\left(-\frac{2\tau^{2}}{\sum_{k}{\left(c_{k}\right)}^{2}}\right), \end{array}$$

where Pr (*Ω*) denotes the probability of event *Ω*.  Corollary 2 is deduced from Theorem 1.

**Corollary 2 .**

Consider a fixed region couple (*R*, *R*′) of *I*, and $\bar{R}$ is the observed gray level average of region *R*. ∀0 < *δ* ≤ 1, we have

(14)
Pr
(|(R¯−R¯′)−E(R¯−R¯′)|≥G12Q(1|R|+1|R′|)ln⁡2δ)≤δ.

The proof can be referred to from [24]. As for the proposed third clustering procedure, region variance is used as the statistical variable instead of mean. The statistical inference inequality for variance is deduced based on the following corollary.

**Corollary 3 .**

Consider a fixed region couple (*R*, *R*′) of *I*, and *φ*_*R*_ is variance of region *R*. ∀0 < *δ* ≤ 1; we have

(15)
Pr
(|(φR−φR′)−E((φR−φR′))|≥G2Q12(h(|R|)+h(|R′|))ln⁡2δ)≤δ,

where *h*(|*R*|) = 1/|*R*| + 1/|*R*|^3^ − 2/|*R*|^2^.

**Proof**

For any region *R*, *φ*_*R*_ = (∑_*i*∈*R*_(*x*_*i*_−*μ*)^2^)/*N* = ∑_*i*∈*R*_ *x*_*i*_^2^/*N* − *μ* ^2^. Suppose we shift the value of the outcome of one r.v. *p* among the *Q*(|*R*| + |*R*′|) possible for the couple regions (*R*, *R*′) by the largest value *G*/*Q*. If *p* ∈ *R*, the new region variance *φ*_*R*_′ is

(16) φR′=1N(∑i∈R/pxi2+(xp+GQ)2) −(1N(∑i∈R/pxi+xp+GQ))2=φR+1|R|(2GQxp+G2Q2)−(1|R|GQ)2−2|R|GQμ→xp=G/Q,μ=G/Q =φR+(1|R|−1|R|2)G2Q2.

|*φ*_*R*_ − *φ*_*R*′_| is subject to a variation of at most *c*_*R*_ = (1/|*R*| − 1/|*R*|^2^)(*G* ^2^/*Q* ^2^). Otherwise, if *p* ∈ *R*′, then *c*_*R*_ = (1/|*R*′| − 1/|*R*′|^2^)(*G* ^2^/*Q* ^2^). We have

(17) ∑k(ck)2=Q2(|R|(cR)2+|R′|(cR′)2)=G4Q2(1|R|+1|R|3−2|R|2+1|R′|+1|R′|3−2|R′|2).

Thus, Corollary 3 can be deduced from Theorem 1.

From Corollary 3, we get our predicate inequality for the third merging procedure: regions *R* and *R*′ should be merged if the following inequality holds; otherwise we should give up merging and go on to the next couple regions to be predicated:

(18) $$\begin{array}{c} \left|\varphi_{R}-\varphi_{R^{\prime }}\right|<\frac{G^{2}}{Q}\sqrt{\frac{1}{2}\left(h\left(\left|R\right|\right)+h\left(\left|R^{\prime }\right|\right)\right)\ln\frac{2}{\delta }}. \end{array}$$

Like Nock and Nielsen did in the classical merging method, we also loosen the restriction to the following inequality [24]:

(19) $$\begin{array}{c} \left|\varphi_{R}-\varphi_{R^{\prime }}\right|<\sqrt{c^{2}\left(R\right)+c^{2}\left(R^{\prime }\right)}, \end{array}$$

where $c\left(R\right)=\left(G^{2}/Q\right)\sqrt{\left(\left(1/2\right)h\left(\left|R\right|\right)\right)\ln\left(\left|R_{\left|R\right|}\right|/\delta \right)}$.

### 3.2. Modified Statistical Region Merging Procedure

There are two clustering procedures for the traditional SRM algorithm. The average gray level is used as the inference variable. The first clustering procedure is based on 4-connexity system with *N* < 2 | *I*| couples of adjacent pixel pairs. The clustering order is set based on the Sobel gradient between the pixel couples. The first clustering procedure results in a primary segmentation map. To handle occlusions, the SRM is run again on the primary map. The MSRM is exhibited based on the procedures above, with the only modification to act on an 8-connexity system with *N* < 4 | *I*| couples of adjacent pixel pairs instead of 4-connexity to avoid block-like clustering result. Hereafter, a third clustering procedure is defined based on the resulting segmentation map. The region variance is used as the inference variable with the inference inequality shown in (19). The merging principle is defined as follows: (1) order the regions of second merging result map by $\bar{R}$ from the darkest to the brightest as $\left\{{\bar{R}}_{1}\leq {\bar{R}}_{2}\leq \cdots \leq {\bar{R}}_{K}\right\}$; (2) begin the merging process from the darkest region couple (*R*_1_, *R*_2_) until (*R*_*s*_, *R*_*s*+1_) cannot satisfy the merging inequality (19), and regions (*R*_1_, *R*_2_,…*R*_*s*_) are clustered as the unchanged class {*uc*}; and (3) merge (*R*_*s*+1_, *R*_*s*+2_,…, *R*_*K*_) as the changed class {*cc*}. The final change detection map is gained after going through the three clustering processes.

As mentioned in [24], the image statistical complexity parameter *Q* is tunable to control the coarseness of segmentation. An intuitionistic choice is to set *Q* as 2^*L*^, where *L* is the number of bits per pixel. However, we find the risk of overmerging for NLR. So we set *Q* = 2^*L*+1^ instead (*L* = 8 in our experiments). Experimental results show the robustness of *Q* for all the experimental data with changes of different kinds and scales.

### 3.3. Summary of the Proposed Merging Method

**Figure 1:**
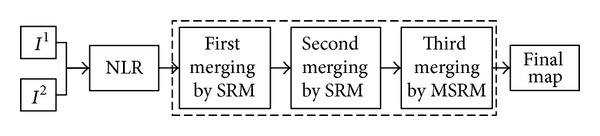
General scheme of the proposed approach.

The proposed region merging method is performed on the NLR DI generated by inequality (11). It composes three steps as shown in the dashed block in Figure 1. For the first merging step based on the traditional SRM, neighbor pixel couples (*p*, *p*′) in 8-connexity system are sorted in increasing order of gradient between (*p*, *p*′) in the corresponding direction. Following the order, *R*(*p*) and *R*(*p*′) are merged if inequality (12) holds, where *R*(*p*) stands for the current region to which *p* belongs. The first merging result is attained after traversing this order once and then used as the input of the second merging procedure. The purpose of the second merging is to handle occluded regions of similar gray levels. Regions of the first merging result are sorted in increasing order of average gray difference $\left|\overset{-}{R}-{\overset{-}{R}}^{\prime }\right|$. Inequality (12) is still taken as the merging predicate. The merging result is used as the input of the third merging procedure where regions are sorted in the order of average gray level $\overset{-}{R}$. The merging predicate is deduced by the difference of region variance as shown in inequality (19). Regions *R* and *R*′ are merged if inequality (19) holds, following the principle in Section 3.2. Until now, we get the final change map consisting of two classes {*uc*, *cc*}.

## 4. Experimental Results

### 4.1. Experiment Design

**Figure 2:**
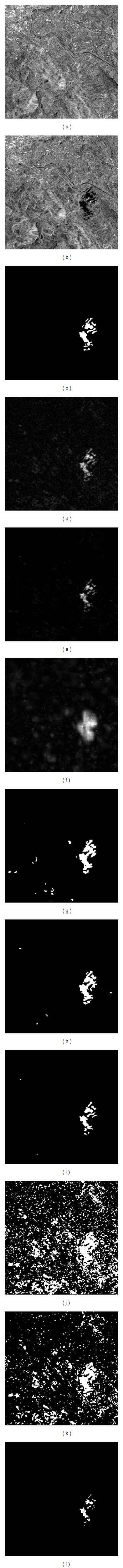
Experimental results on Bern dataset. (a) Image acquired in April 1999, (b) image acquired in May 1999, (c) ground truth, (d) LR DI, (e) NLR DI, (f) CKLD DI, (g) change map by CWT-EM, (h) change map by MRF-FCM, (i) change map by MSRM, (j) change map by LR-GKIT, (k) change map by NLR-GKIT, and (l) change map by GR-HRT.

**Figure 3:**
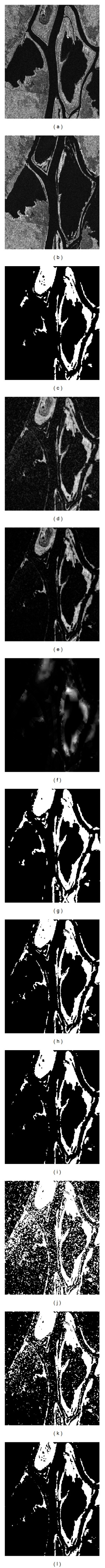
Experimental results on Ottawa dataset. (a) Image acquired in May 1997, (b) image acquired in August 1997, (c) ground truth, (d) LR DI, (e) NLR DI, (f) CKLD DI, (g) change map by CWT-EM, (h) change map by MRF-FCM, (i) change map by MSRM, (j) change map by LR-GKIT, (k) change map by NLR-GKIT, and (l) change map by GR-HRT.

**Figure 4:**
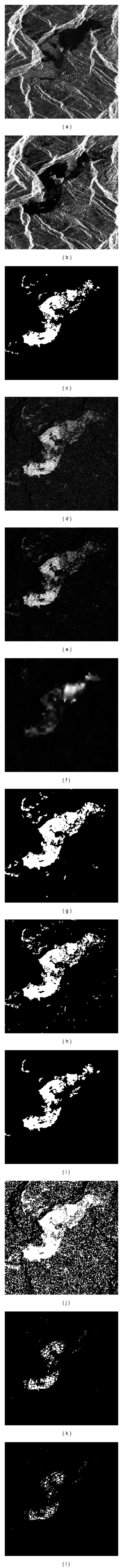
Experimental results on WenChuan Water dataset. (a) Image acquired in March 2008, (b) image acquired in June 2008, (c) ground truth, (d) LR DI, (e) NLR DI, (f) CKLD DI, (g) change map by CWT-EM, (h) change map by MRF-FCM, (i) change map by MSRM, (j) change map by LR-GKIT, (k) change map by NLR-GKIT, and (l) change map by GR-HRT.

**Figure 5:**
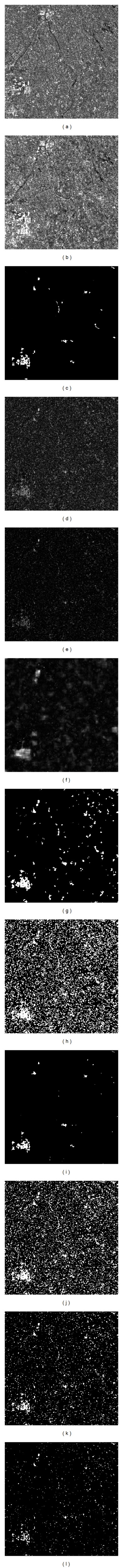
Experimental results on WenChuan Building dataset. (a) Image acquired in March 2008, (b) image acquired in June 2008, (c) ground truth, (d) log ratio DI, (e) Nakagami DI, (f) CKLD DI, (g) change map by CWT-EM, (h) change map by MRF-FCM, (i) change map by MSRM, (j) change map by LR-GKIT, (k) change map by NLR-GKIT, and (l) change map by GR-HRT.

**Figure 6:**
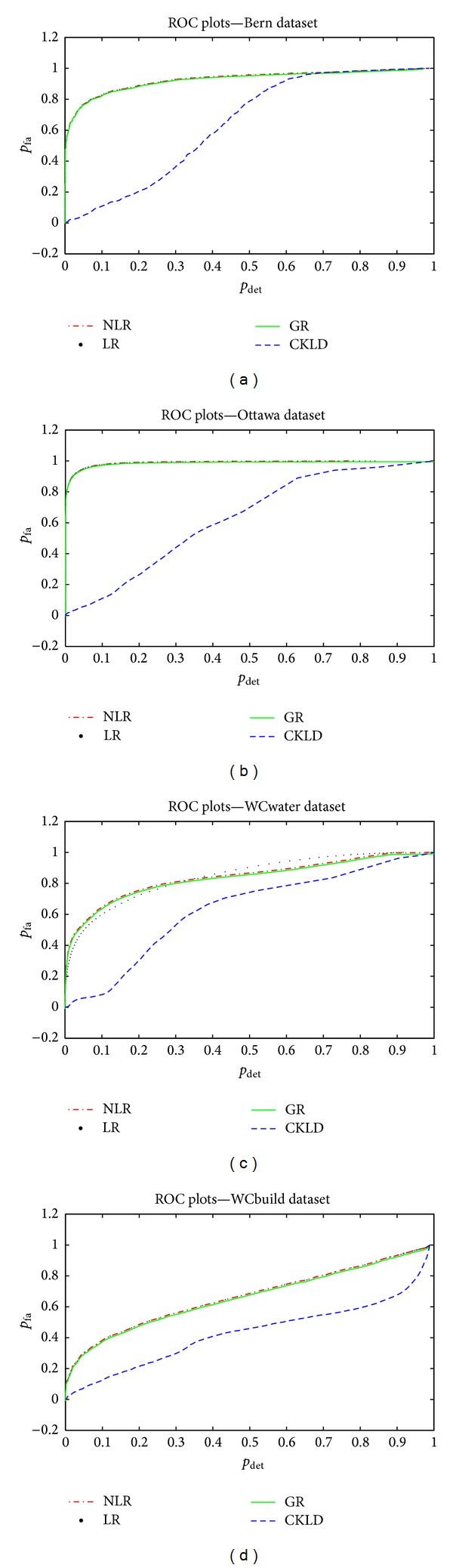
ROC plots comparison between NLR, LR, GLR, and CKLD. (a) Bern dataset, (b) Ottawa dataset, (c) WCwater dataset, and (d) WCbulid dataset.

The proposed method is applied to three different SAR image datasets, including two widely used CD datasets and our own dataset with two selected areas. The first is the Bern dataset shown in Figure 2. It represents two SAR images acquired by the ERS-2 satellite SAR sensor over an area near the Bern city in April and May 1999, respectively. During this time, the Aare valley was flooded. A section size of 301 × 301 is chosen for CD experiment. The second is the Ottawa dataset size of 290 × 350 shown in Figure 3, acquired by Radarsat-1 SAR sensor over the Ottawa city in May and August 1997, respectively. Also a flood happened during this time. Two selected areas of the third dataset are shown in Figures 4 and 5, acquired by the ESA/ASAR sensor over WenChuan area in China in March and June 2008, respectively. During this time, the terrible WenChuan earthquake struck this region. The first WenChuan dataset termed as WCwater consists of changed water regions. The second one termed as WCbuild includes collapsed buildings and new buildings to accommodate homeless residents. The two subsets are both of size 500 × 500. The reference images of the two WenChuan datasets are created by manual thresholding, with the noise spots removed manually. Difference images generated by log ratio, cumulant based KLD, and the proposed Nakagami operator are shown in Subfigures (d), (e), and (f), respectively, in Figures 2–5. In order to evaluate the performance of different DI quantitatively, ROC curves have been generated as shown in Figure 6.

To verify the improvement of CD accuracy of the proposed MSRM algorithm, segmentation method proposed in [8] is applied as a typical thresholding approach termed as CWT-EM which is based on multiscale analysis (by the dual-tree wavelet transform) against speckles. Method in [3] termed as MRF-FCM is used as a typical clustering approach incorporating MRF model to reduce the effect of speckles. Thanks are due to the authors for sharing the implementation Matlab codes on the Internet. Furthermore, two basic and fast threshold based change detection methods proposed in [4, 5] are implemented for the computation efficiency comparison. In [5], the Kittler and Illingworth minimum-error thresholding algorithm is generalized (termed as GKIT) to take into account the non-Gaussian distribution of the amplitude values of SAR images. With the distribution parameter estimated by a feasible technique MoLC (method of log-cumulants), the GKIT exhibits very short computation times. As mentioned in the last paragraph of Section 2, [4] proposes a similar DI generation method where SAR intensity images are used for change detection. Note that SAR image intensity value is the square of amplitude value; thus the experimental images are squared before generating the GR DI. It also presents a straightforward way to determine the segment threshold automatically (termed as HRT). The ratios of DI's histogram at two adjacent gray level values on the right side of the unchanged peak are calculated, and the first point with a ratio less than 1.0 is taken as the threshold. Subfigures (g), (h), and (i) in Figures 2–5 are the change detection final maps of CWT-EM, MRF-FCM, and the proposed MSRM method performed on NLR, respectively. Subfigures (j) and (k) are the CD results by GKIT on LR and NLR, respectively. Subfigure (l) is the final map of HRT method performed on GR.

The Kappa coefficient shown in Table 1 is used to measure the CD accuracy of different methods. It takes both missed detections and false alarms into consideration and hence is an overall evaluation criterion. The Kappa is defined as

(20) $$\begin{array}{c} \text{Kappa}=\frac{pr_{0}-pr_{c}}{1-pr_{c}}, \end{array}$$

with

(21) $$\begin{array}{c} pr_{0}=\frac{N-Fc-Fu}{N}\\ pr_{c}=\frac{Tc+Fc}{N}\times \frac{Tc+Fu}{N}+\frac{Tu+Fu}{N}\times \frac{Tu+Fc}{N}, \end{array}$$

where *N* is the total pixel number of DI, *Tc* is number of correctly detected changed pixels, *Tu* is number of correctly detected unchanged pixels, *Fc* is number of false detected changed pixels, and *Fu* is number of false detected unchanged pixels. A bigger Kappa indicates a better performance. With all the CD methods executed on an Intel (R) Core (TM) i7-2600 @ 3.4 GHz processor implemented by Matlab R2011a, the execution times are also presented in Table 1.

### 4.2. DI Comparison

By observing subfigures (d), (e), and (f) in Figures 2–5, it can be seen that CKLD gives a smooth DI, but it apparently cannot preserve geometrical information. The proposed NLR depresses speckles obviously compared with LR for all the four experimental datasets, which avoids the trouble of despecking. We owe the good performance of NLR to incorporating the prior information of speckles' Nakagami distribution. The phenomena can also be explained in an intuitive way. For any SAR images pair *I* ^1^, *I* ^2^ within [1, *L*], the LR DI would be in the range of [log⁡(1), log⁡(*L*)], while NLR would be in [log⁡(2), log⁡(*L* + 1/*L*)] (for *L* ≫ 1, log⁡(*L* + 1/*L*) ≈ log⁡(*L*)). Assume *r* = *I*_*s*_^2^/*I*_*s*_^1^, and 1 < *r* < *L*; we have

(22) $$\begin{array}{c} f\left(r\right)=\frac{\log\left(r+1/r\right)-\log\left(2\right)}{\log\left(L+1/L\right)-\log\left(r+1/r\right)}-\frac{\log\left(r\right)-\log\left(1\right)}{\log\left(L\right)-\log\left(r\right)}<0. \end{array}$$

It means that for any site *s* on DI, NLR_*s*_ is more biased to the corresponding unchanged side (towards log⁡⁡(2)) compared to LR_*s*_ (whose unchanged side is towards log⁡⁡(1)). Note that each DI is scaled to [1,256] for the proposed MSRM segmentation method. Speckle sites on the NLR would look darker compared with LR, while the changed sites are still bright.

The ROC plot shown in Figure 6 presents quantitative comparison between LR, CKLD, GR, and the proposed NLR. For all the four datasets, the CKLD curves are far below the other three DIs (a similar report can be found in [25]). On the contrary, the LR, GR, and the proposed NLR give almost the same performance (that is why we did not put GR images in Figures 2–5). It is because that the NLR (or GR) can be seen as a monotonically enhanced version of LR by a nonlinear mapping function, which does not modify the performance of the detector in terms of ROC.

Five segmentation methods mentioned above are performed on both LR and NLR with the exception that HRT is performed on GR and NLR. The resulting kappa coefficients are shown in Table 1. There is little difference between the kappa of NLR and LR for the CWT-EM and MRF-FCM method, which take multiscale or local statistical correlation information into consideration and consequently are more robust to different DI. Owing to a better statistical separability between the unchanged and changed regions on NLR (compared with LR), GKIT which only makes use of the gray distribution of the whole image is able to find a better threshold and thus gives a higher kappa. The improvement can be seen by comparing subfigures (j) and (k) in Figures 2–5. For HRT method, NLR performs better than the original GR on Bern and WCwater datasets while worse than GR on Ottawa and WCbuild datasets. We blame this unstable phenomenon on the fact that the HRT only relies on the ratios of DI's histogram (which is unpredictable) at two adjacent gray level values to decide the global threshold. In contrast, NLR shows better performance than LR for the proposed MSRM method on all datasets, which is also superior to the performance of CWT-EM and MRF-FCM on LR. Even though the ROCs show little difference between NLR and LR (GR), but with a bigger contrast between the unchanged and changed pixels, a better performance can be achieved by the region merging classification method.

### 4.3. Segmentation Methods Comparison

We can see that the performance of CWT-EM method is quite stable on the four experimental data with changes of different characteristic. Most of the changes are successfully detected despite the high false alarm rate caused by the residual speckles. The kappa coefficients shown in Table 1 also indicate that the CWT-EM method gives medium but stable performance. The MRF-FCM method performs quite well for the first three data with changed water area which show good contrast between the changed and unchanged pixels. But the performance is very poor for the WCbuild dataset which is interrupted by more severe speckles; also the contrast is very low. The proposed MSRM method gives better performance than the other four methods with higher kappa coefficients for all the experimental datasets, especially for the fourth dataset, on which the MRF-FCM method is infeasible as a result of falling into a local optimum. GKIT shows bad performance even for the Bern and Ottawa datasets, while the HRT performs very well on Bern and Ottawa but is poor for the WC datasets. The two approaches are presented here mainly for computation efficiency comparison.

The last column in Table 1 shows execution time of different methods on the four datasets. The CWT-EM method provides relatively lower complexity compared to MRF-FCM. The execution time of MRF-FCM is quiet longer than the other methods and varies from scene to scene due to the iterative computation. The proposed MSRM method also depends on the complexity of DI. The more complex it is, the more numerous the regions are in the first merging results and the more time will be taken in the second and third merging procedure. For the Bern and Ottawa datasets which are quite simple, it takes about 2 seconds, which is less than one-tenth of the DT-CWT. For the WCwater dataset, the execution time is 4.90 s, which is one-fifth of the DT-CWT. For the WCbuild dataset with changed buildings of small area and severe speckles, the MSRM execution time is 6.75 s. It is a little longer but still much faster than the DT-CWT and MRF-FCM. The GKIT and HRT give amazingly short execution time by finding a global threshold simply relaying on the image histogram. The GKIT final maps contain plenty of false alarms caused by inappropriate thresholding. In order to get a better performance, the authors apply gamma-MAP despeckling filter to DI. Unfortunately, the despeckling process prolongs the execution time significantly. One iteration of a 7 × 7 gamma-MAP procedure on Bern DI (300 × 300 pixels) costs 6 s on our processor (16 s on WenChuan dataset). The despeckling deprives GKIT of its strength (time efficiency). Similar situation is found in the HRT method. Although HRT generates good CD results on the Bern and Ottawa datasets, it does not work well on the WenChuan dataset. The authors also suggest applying frost filter on images when noticeable speckles are present. If we take despeckling procedure into consideration, the proposed MSRM method is still the fastest one among the five approaches. Besides, it outperforms the others in terms of CD accuracy.

## 5. Conclusions

A novel fast SAR image change detection method using Bayesian approach based difference image and modified statistical region merging has been presented in this paper. Two novel methodological contributions characterize this work. Firstly, a new DI is exhibited through the Bayesian decision theory incorporating the prior of speckles' Nakagami distribution. This new DI is superior to the familiar log ratio DI in the speckle depression effect and outperforms the CKLD in terms of geometrical information preservation. Secondly, based on the traditional SRM method, a new clustering procedure is introduced with the region variance as the statistical inference variable to control the clustering ending procedure. Due to the avoidance of despeckling in the DI generation procedure and by taking advantage of the fast implementation of MSRM, both the change detection accuracy and operation efficiency of the proposed method are improved significantly compared with the state-of-the-art related methods.

## Figures and Tables

**Table 1:** Kappa coefficient and execution time.

Datasets	Methods	Kappa (NLR)	Kappa (LR, GR for HRT)	Execution Time (s)
Bern	CWT-EM	0.699	0.604	15.63
	MRF-FCM	0.779	0.763	22.52
	GKIT	0.182	0.090	0.51
	HRT	0.842	0.641	0.08
	**MSRM**	**0.844**	**0.762**	**1.48**

Ottawa	CWT-EM	0.825	0.830	14.57
	MRF-FCM	0.915	0.920	75.62
	GKIT	0.699	0.445	0.68
	HRT	0.914	0.937	0.02
	**MSRM**	**0.928**	**0.906**	**1.93**

WCwater	CWT-EM	0.798	0.721	23.95
	MRF-FCM	0.856	0.887	121.04
	GKIT	0.373	0.285	0.87
	HRT	0.373	0.209	0.07
	**MSRM**	**0.902**	**0.855**	**4.90**

WCbuild	CWT-EM	0.464	0.393	24.02
	MRF-FCM	0.063	0.054	196.68
	GKIT	0.243	0.092	0.83
	HRT	0.278	0.370	0.08
	**MSRM**	**0.725**	**0.667**	**6.75**
